# Thermodynamic evaluation of viscosity behavior for CaO–SiO_2_–Al_2_O_3_–MgO slag systems examined at the temperatures range from 1500 to 1700 °C

**DOI:** 10.1038/s41598-023-41404-x

**Published:** 2023-09-28

**Authors:** Augusto Lachini Pereira, Julio Aníbal Morales Pereira, Wagner Viana Bielefeldt, Antônio Cezar Faria Vilela

**Affiliations:** Department of Metallurgy (DEMET), Federal University of Rio Grande do Sul (UFRGS), Porto Alegre, Brazil

**Keywords:** Engineering, Materials science

## Abstract

This work showed an application of computational tools to understand systematically the behavior of viscosity on CSAM systems relevant to industrial uses. Consequently in this study, the viscosity experimental data obtained from the literature were compared with the thermodynamic calculated results via the software FactSage v.7.3 for melts in CaO–SiO_2_–Al_2_O_3_–MgO slag system with the range of compositions slags cover 0–100 wt% CaO, 0–100 wt% SiO_2_, 0–100 wt% Al_2_O_3_ and 0–15 wt% MgO at temperature ranges of 1500–1700 °C. Using open-source software in Python, the results of viscosity, liquid, and solid fraction of the slag, as a function of composition and temperature, are represented by multiple color maps and by iso-viscosity contours. The results of the viscosity values indicated that the effect of all the oxides in the CSAM slag system follows the well-known behavior trend observed in the literature. Viscosities of the slag were found to increase with increasing SiO_2_ contents and decrease with increasing basicities (high CaO). The increase in Al_2_O_3_ content increases the viscosity values. An increase of 0–15% MgO depolymerized the slag melt and decreases the viscosity. However, above 5% MgO content occur a decrease in the liquid zone (single phase) and a liquid fraction (two-phase region) of the slag. For a constant MgO concentration, the increase in temperature generates an expansion of low-viscosity zones associated with an increase in the liquid phase of the slag. From the comparison between the calculated and experimental viscosities data keeps up within 30% average relative deviation (Δ), the predictions are considered acceptable for viscosity in the CSAM slag system at high temperatures.

## Introduction

Viscosity is one of the most important physical properties in many industrial applications, for example, steelmaking slags. In general, it varies over a wide range of values depending on chemical composition and temperature. A better fluidity of slag (low viscosity slag) impacts the steel manufacturing process such as reaction kinetics between the slag and the liquid steel, gas permeability, and specifically its cleanliness^1–8^.

Viscosity values can vary greatly depending on chemical composition and temperature. According to Bale et al.^9^ there is a strong relationship with the silica network. Of the various viscosity models available in the literature, the FactSage program applies the Modified Quasichemical Model^10,11^. The model can then successfully predict viscosities in multicomponent systems^12,13^.

There are articles in the literature^14^ that compare the viscosity values calculated by thermodynamic programs with experimental data from the laboratory. Even if the viscosity models are validated with experimental data, work on specific oxide systems is important to deepen the understanding and verify eventual knowledge gaps. Rocha et al. compared results calculated via FactSage with experimental data and the mean deviation was 23.61%.

Concerning the presence of second-phase solid particles on slags, the paper of Saigo et al.^15^ focuses on the viscosity prediction problem in steelmaking and proposes Einstein–Roscoe regression (ERR), which learns the coefficients of the Einstein–Roscoe equation and is able to extrapolate to unseen domains. In experiments using the viscosity measurements in a high-temperature slag suspension system, ERR is compared favorably with various machine learning approaches.

Slag optimization from the quaternary system CaO–SiO_2_–Al_2_O_3_–MgO has key importance as part of the secondary refining of diverse grades of special steels. On focus to improve the absorption ability of slag for non-metallic inclusions and ensuring refractory protection^1,2,16–29^.

Therefore, the viscosity measurement process is considered expensive, the cost-effectiveness does not favor the measures at high temperatures^30^. Alternatively, it is possible to apply mathematical models to obtain viscosities for a given range of chemical composition and temperature of slags^1,31–41^.

One of the well-known simulation tools is computational thermodynamics. Computational thermodynamic calculations are used to understand many phenomena that occur during the processing of liquid steel, for which many reactions may occur. FactSage is a commercially available software employed to simulate various metallurgical processes. Because of its extensive databases, is capable of calculating phase diagrams and phase equilibrium conditions for multi-component systems^42–48^.

In the present study from experimental data collected through several published studies, were calculated the viscosities of liquid and partially liquid (liquid + solid) slags of the CaO–SiO_2_–Al_2_O_3_–MgO (CSAM) slags system at temperatures 1500–1700 °C. The slag properties were represented in the form of color map plots and iso-viscosity contours. It is a novelty in terms of visualization of viscosity values because this paper shows not only the liquid viscosity but also the liquid + solid viscosity according to the Roscoe–Einstein equation.

In secondary steel refining slags, FeO contents lower than 5% are expected, in order to increase the capacity to remove sulfur and non-metallic inclusions. Due to the low contents, and in order to carry out a systematic study in the quaternary system CaO–SiO_2_–Al_2_O_3_–MgO, FeO was not considered in this study.

We consider the innovative character of the article because it represents in ternary systems not only the viscosity of the liquid but also the effective viscosity of slags in the CaO–SiO_2_–Al_2_O_3_–MgO system. We did not find works in the literature with this approach of displaying effective viscosity data. It also made a great effort to compare the results of calculated liquid viscosity with experimental data of slags in the CaO–SiO_2_–Al_2_O_3_–MgO system at temperatures of 1500–1700 °C.

## Methodology

**Figure 1:**
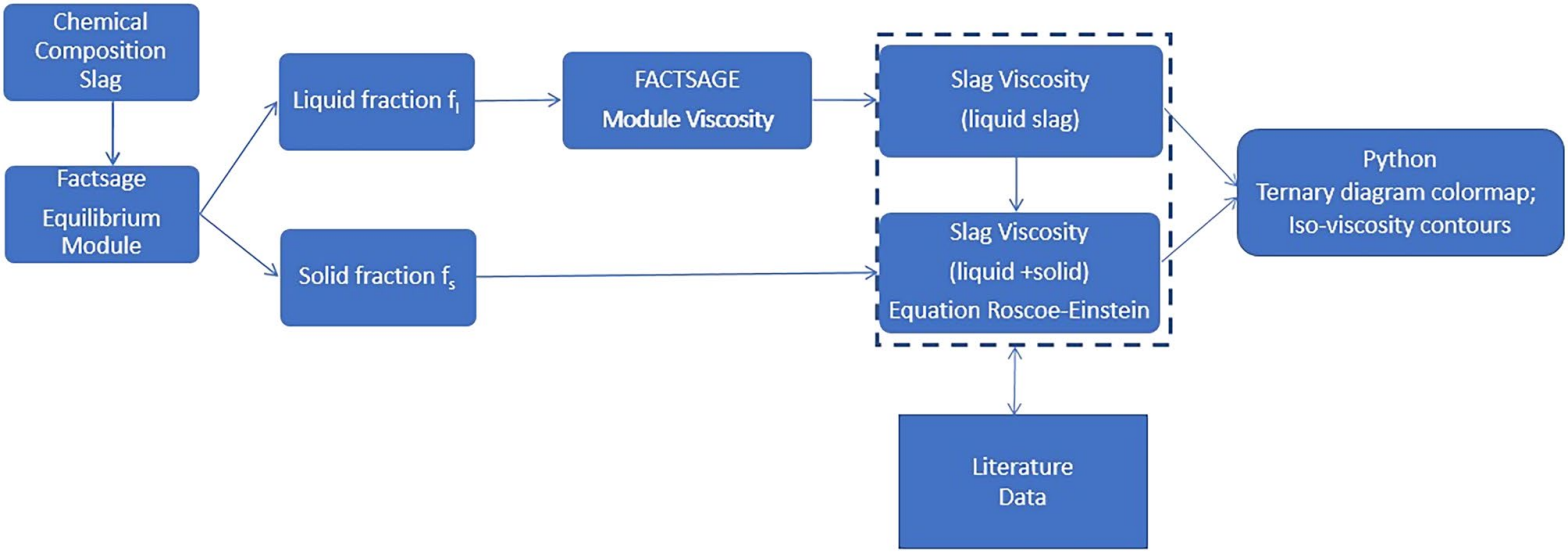
Flow chart of the computational process in this work.

Figure 1 shows the flow chart of the adopted computational procedure applied in this study.

Figure 1 shows the computational procedure that consists of the determination of solid/liquid fractions and performing “liquid viscosity” calculations of slags using the FactSage software. Following the Einstein-Roscoe equation is possible to perform calculations of the “effective viscosity” in a multiphase system in which the slag phase contains a solid fraction. Finally, carried out the construction plot for each chemical composition, temperature, liquid/solid fraction, and calculations of iso-viscosity curves. The thermodynamic calculated results via the software FactSage were compared with the viscosity experimental data obtained from the literature.

Table 1 shows the chemical composition range for each CSAM slag system used in the viscosity calculations.

**Table 1:** Composition ranges of slags to calculate viscosity. (mass %).

Temperature °C	MgO	Phase			CaO		SiO_2_		Al_2_O_3_		Count
		Mixture	Liquid	Solid	Min	Max	Min	Max	Min	Max	
1500	0	x			0	98	0	98	0	98	739
			x		0	60	0	100	0	80	553
				x	0	100	0	34	0	100	34
	5	x			0	93.1	0	95	0	93.1	792
			x		0	53.2	0	89.3	0	58.9	503
				x	0	95	0	34.2	0	95	31
	10	x			0	88.2	0	90	0	88.2	902
			x		0	46.8	0	84.6	0	52.2	394
				x	0	90	0	36	0	90	30
	15	x			0	83.3	0	85	0	83,3	925
			x		0	44.2	32.3	81.6	0	35.7	371
				x	0	85	0	35.7	0	85	29
1,600	0	x			0	98	0	96	0	98	545
			x		0	60	0	100	0	82	749
				x	0	100	0	34	0	100	32
	5	x			0	93.1	0	95	0	93.1	596
			x		0	57	0	91.2	0	66.5	702
				x	0	95	0	34.2	0	95	28
	10	x			0	88,2	0	90	0	88.2	736
			x		0	48.6	0	88.2	0	57.6	566
				x	0	90	0	32.4	0	90	24
	15	x			0	83.3	0	74.8	0	83,3	853
			x		0	44.2	27.2	85	0	45.9	453
				x	0	85	0	30.6	0	85	20
1700	0	x			0	98	0	80	0	98	441
			x		0	62	0	100	0	84	853
				x	0	100	0	34	0	100	32
	5	x			0	93.1	0	95	0	93.1	437
			x		0	58.9	0	93.1	0	74.1	867
				x	0	95	0	34.2	0	95	22
	10	x			0	88.2	0	84.6	0	88.2	545
			x		0	50.4	0	90	0	61.2	761
				x	0	90	0	32.4	0	90	20
	15	x			0	83.3	0	74.8	0	83.3	713
			x		0	45.9	0	85	0	57.8	593
				x	0	85	0	30.6	0	85	20
Total											15,911

The chemical compositions adopted in this work covered all the single-phase liquid regions and some two-phase (liquid + solid) regions. The spacing between each composition is in the order of 2 wt%. This variation is related to the size of the stability phase region. A total of 15,911 chemical compositions were used to generate a specific viscosity value. This current work adopted the binary basicity, i.e. the C/S—a ratio of components in mass percentages, (C = CaO and S = SiO_2_)^22^. High basicity slags are slags with C/S > 1 and Low basicity slags, are slags with C/S < 1. An important limitation of the basicity index is to classify oxides between basic and acidic. Furthermore, the ability to form or break networks is different between oxides. CaO has a greater ability to break networks than MgO, for example. Al_2_O_3_ has an amphoteric character; its basic or acidic character depends on its concentration and the presence of other oxides. For these reasons, the simplest index of basicity was chosen.

### Thermodynamic calculations

The commercial software FactSage version 7.3 was used for the thermodynamic calculations performed in this study. Its Database covers most of the slag system and temperature ranges^42,43,49^.

#### Solid/liquid fraction of slag

The selected databases were FactPS (stoichiometric pure substances) and FToxid (for oxides and sulfur). On the Equilib module, the initial chemical composition of the selected slag system (CaO, SiO_2_, Al_2_O_3_, MgO) is fed, obtaining the results fractions formed (liquid and solid) for a given temperature of 1773 K (1500 °C**)** to 1973 K (1700 °C). Each chemical composition of the liquid phase resulting is then used as input data for the calculation of the viscosity in the Viscosity module. Phase diagram module for computation of phase equilibria in the quaternary phase diagram of CaO–SiO_2_–Al_2_O_3_–(0–15%) MgO slag were also used^14,17,24,25,27,28,50^.

#### Viscosity calculations

From the Viscosity module (Melts database) is calculated the liquid slag viscosity (hl)^14,17,24,25,27,28,50^. This software uses the modified Quasichemical model (MQM), to describe the thermodynamic behavior of the liquid slag, which used quadruplet data detained from MQM, to calculate the viscosity of liquid^42,46–48^.

To estimate the "Effective viscosity" (when some solid fractions are present), the following Eq. (1) proposed by Roscoe-Einstein was adopted^2,35,51^.

1 $$\upeta =\upeta _{l} - (1 -\uprho {\text{f}})^{( - 2.5)}$$

In Eq. (1), η_l_ represents the viscosity of the liquid, and f the solid fraction slag, ρ represents a solid interaction parameter that, in this study, considering a dilute concentration of spherical particles of different sizes, is assumed to be equal to 1^43,44^. The value of the exponent in Eq. (1) is associated with the geometric shape of the solid particle^2,35,51^. The application of Eq. (1) is limited to the assumption that solid particles with low solubility in the liquid slag are homogeneously distributed^35^.

The original Einstein-Roscoe equation used 'volume fraction of solid' instead of 'solid fraction' and a correction term for morphology, but all these values are not very well-known for general solids. It was used the solid fraction (wt fraction) for this equation as an approximation.

Finally, all color map ternary plots and iso-viscosity contours were created using open-source software in Python^52,53^. The viscosity data, liquid/solid fraction (as a function of chemical composition and temperature), were generated on images Colormaps/Heatmaps 2D plot, using NumPy and matplotlib library^54–56^. The iso-viscosity contours were generated by linear interpolation of the viscosity values of the slags.

### Accuracy of factsage viscosity calculations

The thermodynamic calculated results via software FactSage were compared with the viscosity experimental data obtained from the literature^14,18–20,31,33,34,37–41,57–59^ (see Fig. 1). Table 2 shows the composition ranges of all the reference source data of the CSAM system.

**Table 2:** Composition ranges of slags of the reference sources, (mass%).

System	Temperature (°C)	MgO	SiO_2_	Al_2_O_3_	CaO
SiO_2_–Al_2_O_3_–CaO	1400–2083	0	0–70	0–54	10–55
SiO_2_–Al_2_O_3_–CaO-MgO	1372–1720	0–38	10–65	0–30	1.5–55

The performance analysis of the viscosity calculations through FactSage with the reference viscosity data was evaluated by the Percent Error ($\delta$) and average relative deviation (Δ), for N measurements of viscosity, using Eqs. (2) and (3) respectively

2 $${\delta }_{i}=\frac{{\eta }_{calculated}-{\eta }_{Reference}}{{\eta }_{Reference }} \times 100\%$$

3 $$\Delta =\frac{1}{N} \sum_{i=1}^{N}{\delta }_{i} \times 100\%$$

## Results and discussion

**Figure 2:**
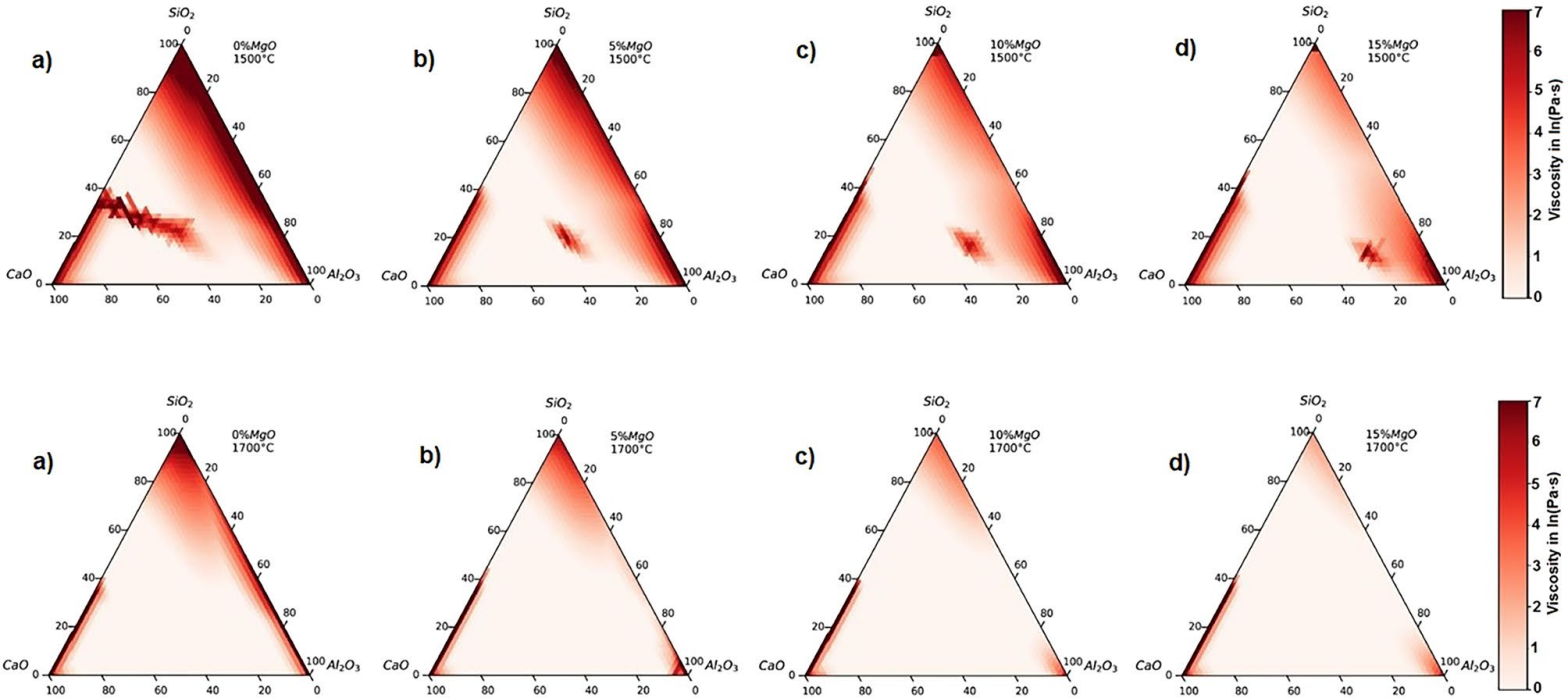
Slag viscosity as a function of chemical composition at temperatures of 1500 and 1700 °C.

**Figure 3:**
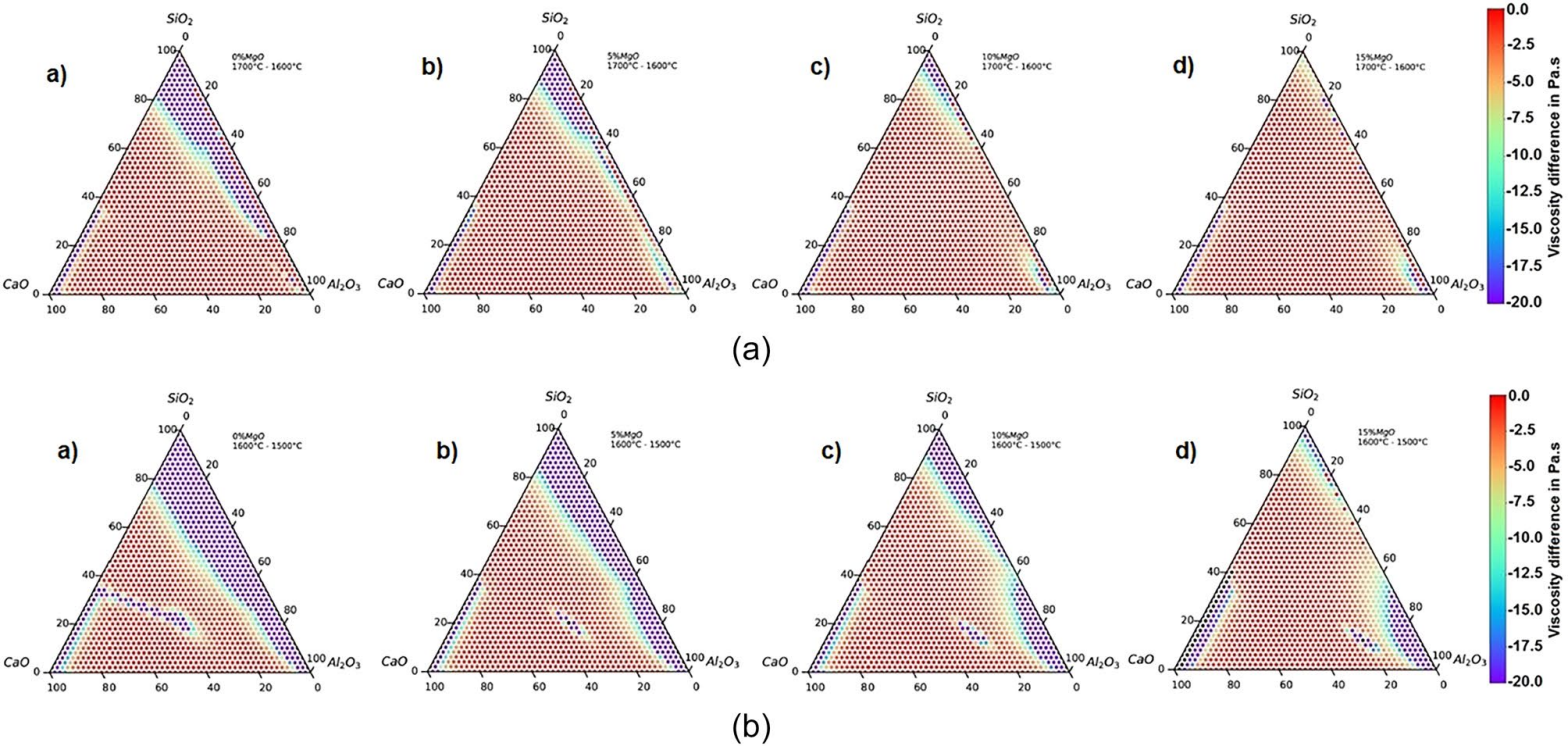
Viscosity difference (Pa s) values in CaO-SiO_2_-Al_2_O_3_-(0–15 mass% MgO) slag system: (**a**) 1700–1600 °C and (**b**) 1600–1500 °C.

**Figure 4:**
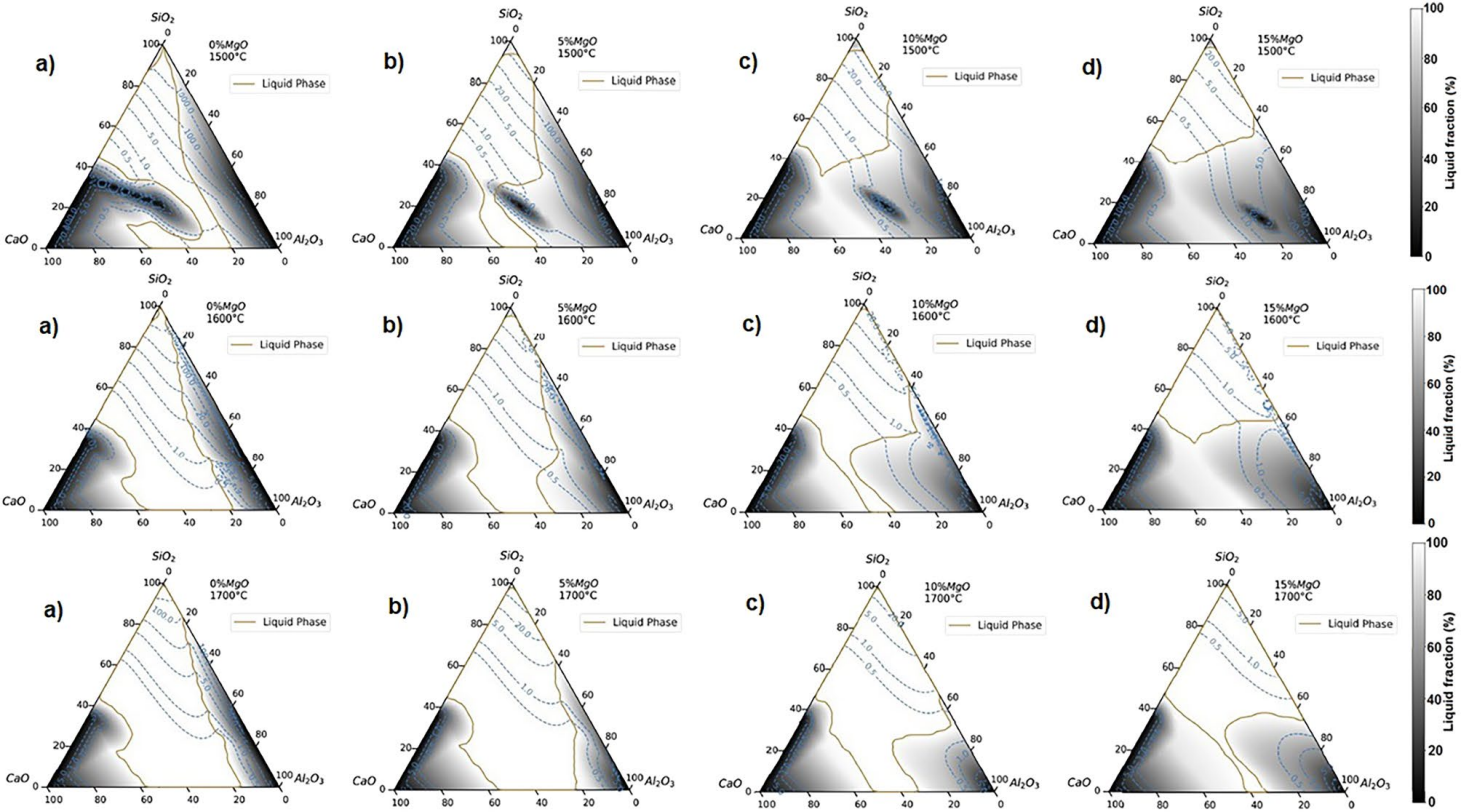
Liquid/solid fraction and iso-viscosity contours for the CSAM system at different content of MgO at temperatures of 1500, 1600, and 1700 °C.

**Figure 5:**
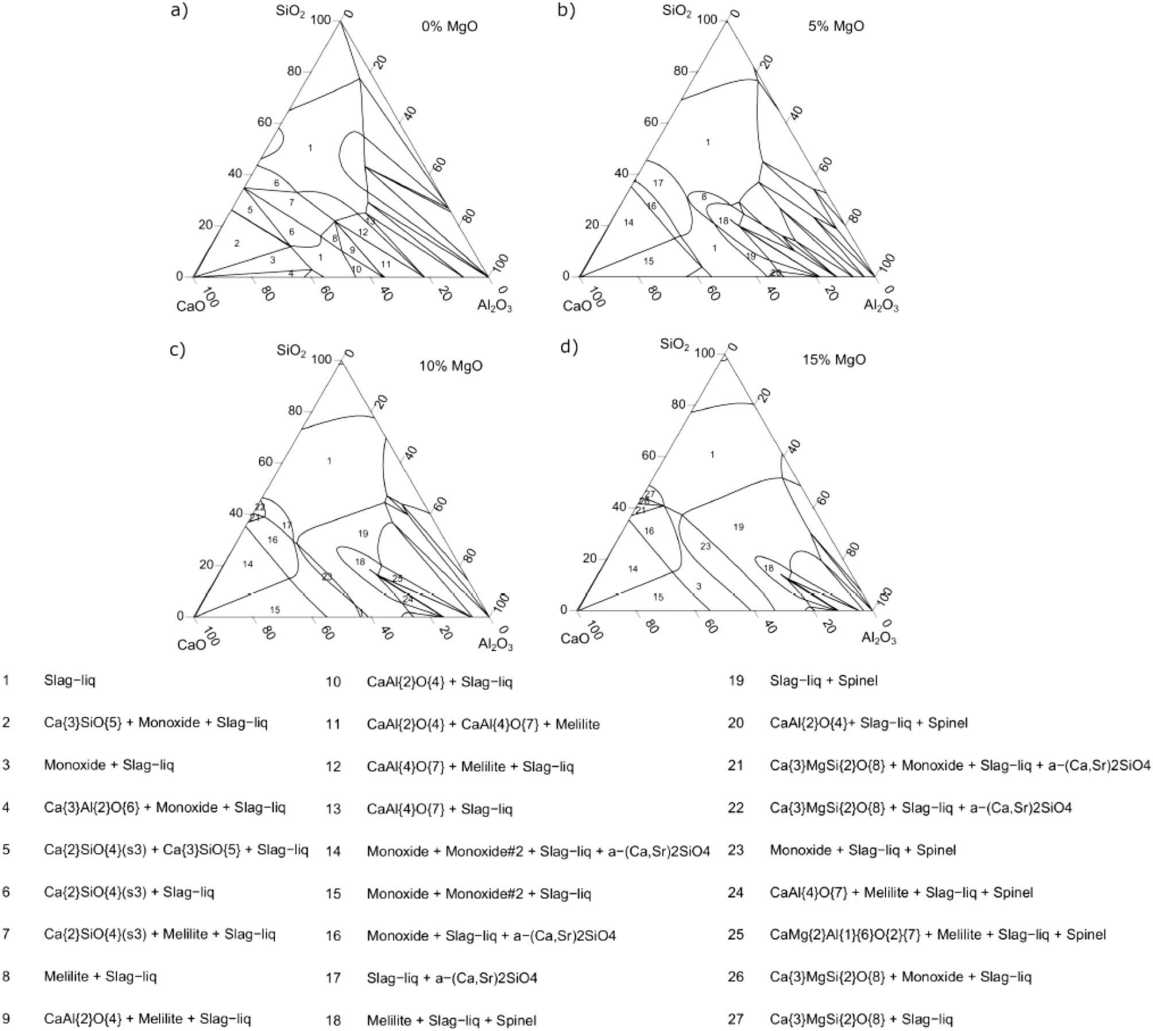
Phase equilibria distribution of CaO–Al_2_O_3_–SiO_2_–MgO slag system at 1500 °C with 0–15% MgO.

Figures 2, 3, 4, 5 show the result of thermodynamic calculation of slag viscosity and liquid/solid fraction accomplished with iso-viscosity contours at temperatures 1500 °C [1773 K], 1600 °C [1873 K] and 1700 °C [1973 K].

### Viscosity

Figure 2 shows the chemical composition dependence on the viscosity at temperatures of 1500 and 1700 °C. The figures show a viscosity scale in red coloration for eye guidance. Slags that show the reddest coloration are more viscous, and on the other hand, the slags with lower viscosities are shown in white coloration. A more detailed discussion will be given in Section “Liquid/solid fraction and iso-viscosity curves” about the areas of red coloration located internally in the ternary diagram at a temperature of 1500 °C and which disappear at a temperature of 1700 °C.

For an initial content of 0% MgO, Fig. 2, a tendency of high viscosity is observed in the regions close to the corners and along the binary axes of the (CaO–SiO_2_; SiO_2_–Al_2_O_3_) diagram. On the other hand, regions of low viscosity cover the base of the diagram (binary CaO–Al_2_O_3_ axes) and the central region. From the experimental results of other researchers^19,20,31,34,60–65^ reported for the binary, ternary, and quaternary slag systems, the impact of each oxide component in the composition range of slag was studied to conclude that with decreasing SiO_2_ content the viscosities always decrease and vice versa. The increase of the SiO_2_ content in the molten slag leads to an increase in the number of network units, hence the polymerization will strengthen the network structure of the slag (Si–O bonds) increasing the viscosity^31,34,60^.

With increasing MgO content from 0 to 15 wt%, Fig. 2, 1500 and 1700 °C, the viscosity of the slag melt decreases. This implies that MgO behaves as a network modifier. Therefore, the effect of MgO addition on viscosity is more effective at low basicities and tends to decrease at high basicities. The fact that MgO is considered a weaker depolymerizer than CaO explains in terms of structural change that increasing basicity is more effective than increasing MgO content in reducing slag viscosity^20^. On the other hand, a high content of basic oxides (CaO and MgO) leads to an increase in viscosity, liquidus temperature (melting), and a reduction in the liquid area of the slag^16,23,31^.

### Temperature

Figure 3 shows the viscosity of CaO–SiO_2_–Al_2_O_3_–(0–15 mass %MgO) slag systems. The first set of ternaries (Fig. 3a) comprises temperatures between 1700 and 1600 °C and the second set of ternaries (Fig. 3b) comprises temperatures between 1600 and 1500 °C. Both are visualized in a rainbow color ternary plot.

In both cases, the figures show the "viscosity difference" of slags in negative values. It is expected because with increasing temperature the viscosities will decrease, so subtraction will result in negative values.

Figure 3a shows that variation of viscosity in the higher temperature range [1973 K (1700 °C) − 1873 K (1600 °C)] is smaller when compared to Fig. 3b [1873 K (1600 °C) to 1773 K (1500 °C)]. According to results from other researchers^18–20,61^, higher temperatures decrease the viscosity of the slag, where the excess thermal energy can provide sufficient energy to break the existing complex network structures decreasing the viscosity. With the increase of MgO from 0 to 15 wt%, there was no significant decrease in viscosity in the higher temperature range [1973 K (1700 °C) − 1873 K (1600 °C)]^20^. Several researchers^13,27,54^ have demonstrated these fluctuations of viscosity about temperature change from the calculation of activation energy for viscous flow. The decrease of activation energy indicates the reductions of the energy barrier for viscous flow and the higher, the greater dependence on temperature viscosity. For the low-temperature range of 1600–1500 °C, another factor to consider is the possible appearance of the breakpoint temperature of slag (the temperature at which there is a significant viscosity increase attributed to the precipitation of solid phases). The break temperature is below of temperature liquidus and generally, breakpoint temperature increases with the increase of basicity and MgO content^20,34,35^.

Experimental results are opposite to the previous ones obtained by FENG et al. (2019)^60^ investigated the effect of CaO/SiO_2_ on break point temperature in slag systems containing TiO_2_. This is, breakpoint temperature decreases with increasing basicity.

### Liquid/solid fraction and iso-viscosity curves

Figure 4 shows the assemblage of the liquid/solid fraction and iso-viscosity curves for the CSAM system at different content of MgO at temperatures of 1500, 1600, 1700 °C. The figures exhibit a black to gray and white scale, representing the solid, liquid/solid mixture, and the liquid region of the slag delimited by a line in gold coloration, and the viscosity iso-curves in dashed lines in blue color.

The analysis of Fig. 4 shows that an increase in MgO content decreases the viscosity combined with a gradual reduction in the liquid region of slag^18,20,23,30^. Also is possible to see the increased liquid fraction (grey areas) in the areas of the diagram that were previously black (presence of solid area). The liquid fraction was about 60% (with 40% of the solid fraction) at 1773 K (1500 °C) for about 80% liquid fraction (with 20% of the solid fraction) above 1873 K (1600 °C). It is also observed that the low viscosity area (0–1 Pa s) is much larger or predominant in slags with high MgO contents and for higher temperatures, as confirmed by the experimental data in the literature^18,20,31^.

Ma et al. 2014^23^ studied slag optimization for the absorption of Al_2_O_3_ inclusions in bearing steels and CaO–Al_2_O_3_–SiO_2_–MgO quaternary systems at temperatures of 1873, 1773, and 1673 K. The authors noted the gradual increase in the liquid region of the slag, with decreasing MgO content from 10 to 4% and increasing slag melting temperature, with increasing %MgO content above 5%. However, in this present study, for better control of the slag composition, the %MgO content was set between 5 and 10% MgO.

Regarding the crystallization behavior of molten slags, Fig. 4a at 1500 °C, shows the area of 40–70% CaO, 20–40% SiO_2_, and 0–40% Al_2_O_3_, consisting of a large fraction of solid that moves as the increase in MgO content increases. In addition, where the iso-viscosity curves are distorted and have a higher viscosity value.

To explain this effect, Fig. 5 (calculated by FactSage) shows the phase equilibria distribution of the CaO–Al_2_O_3_–SiO_2_–MgO slag system at 1500 °C with 0–15% MgO, respectively.

By comparing Figs. 2, 5 at 1500 °C, it can be observed the existence of regions (7, 8, 12, 18, and 25), which correspond to the crystalline phase known as Melilite ((Ca_2_(Al, Mg, Fe^2+^), (Si; Al)_2_ O_7_) being responsible for the increase the viscosity of the slag system. Melilite is a binary solid solution consisting of Gehlenite [Ca_2_Al_2_SiO_7_]–Akermanite [Ca_2_MgSi_2_O_7_]. The melting point of Gehlenite is 1593, 1454 °C for Akermanite^58,65–69^.

The increase of MgO concentration stabilizes the Melilite phase to lower CaO, SiO_2_, and high Al_2_O_3_ contents. The Melilite phase will contain mostly Akermanite, which has a lower melting point. Thus slags with higher MgO concentration will limit the solid fraction effect induced by the melilite phase formation until disappear with increasing temperature as seen in Fig. 2 at 1500 °C^68^. Kim et al.^18^ observed that with the effect of increasing MgO, the phase transformation from melilite to spinel occurs, which increases the activation energy for viscous flow, which increases the viscosity of the slag. The temperature range studied by Kim et al. was [1773 K (1500 °C) − 1673 K (1400 °C)].

### Accuracy of the viscosity model

**Figure 6:**
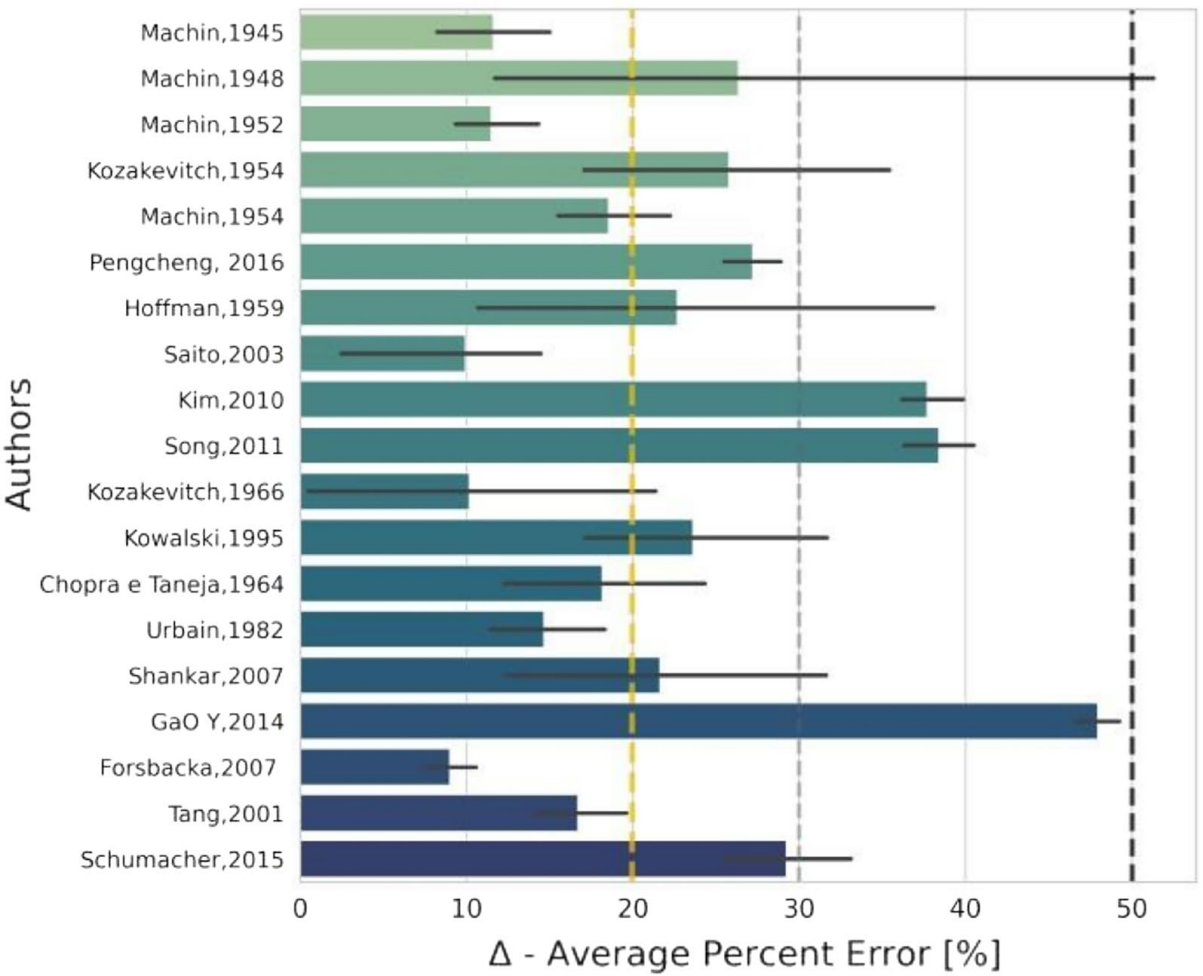
The average relative error was calculated from FactSage and measured viscosity comparison for each reference.

Figure 6 shows experimental results of molten slag viscosities compared to FactSage model results. Also shows the average relative error between the measured reference viscosity data and calculated viscosities.

In the comparative analysis, the average relative error of viscosities is about 20%. According to (MILLS et al., 2001)^38^, the viscosity measurements are subject to experimental uncertainties, such as the effect of crucible materials, temperature differences between the thermocouple reading, and the actual temperature of the melt. Because of this in some cases, will be considered by up to 30%, as proposed by^14,38,47^.

In this context, it can be said that the obtained results in terms of relative deviation, validate the quality of the viscosity model implemented in FactSage in the case of multicomponent slag systems. Thus, the model can be used to optimize aspects of steelmaking processes. In estimating the viscosity of fully liquid BOF slag after the addition of Al_2_O_3_/SiO_2_, the FactSage viscosity model has shown the smallest error (29.10%) when compared to other viscosity models^70^.

## Conclusions

In summary, FactSage 7.3 was employed to analyze the behavior of slags for the CSAM systems at 1500, 1600, and 1700 °C. The Roscoe-Einstein was used to predict the viscosity of slags containing solid fractions. The calculated data were compared with the experimental data collected in many references cited in this work. About 570 experimental data were collected to compare the data with the calculated values, we propose an accurate representation of iso viscosity curves applied for many industrial slags. The following main conclusions can be drawn from this work:

A comparison between the viscosities data collected from the literature with the viscosities calculated by FactSage shows a mean percent error lower than the 30% mean percent error typically obtained during viscosity measurement.

By the analysis proposed, it is possible to visualize that the effect of the MgO content (0–15 wt%) decreases the viscosity. As a further observation, increasing MgO showed an increase in the size of the low viscosity zones (0–1 Pa s).

Al_2_O_3_ showed amphoteric behavior, increasing viscosity at 15–25 wt% and decreasing viscosity at high concentrations. Although it is possible to notice the increase of the solid fraction with alumina higher than 40 wt%, this effect intensified when the system has MgO content greater than 5 wt%.

The FactSage software was a great help in the creation of pseudo-ternary systems with iso-viscosity curves. This is a useful representation for steelmakers, considering the slag designing process, aiming at obtaining the best conditions for steel refining.

## Data Availability

The data supporting the results reported in this study can be found in the respective references used. However, the dataset generated during and/or analyzed during the current study is made available by the authors upon reasonable request and with permission from Augusto Lachini Pereira.
